# Technology‐enabled integrated care pathway (Tech‐ICP) for behavioral and psychological symptoms of dementia in long‐term care

**DOI:** 10.1002/alz70857_105829

**Published:** 2025-12-25

**Authors:** Suzanna Apostolovski, Jordanne Holland, Amer M. Burhan, Mary Chiu, Peter Derkach, Anuroop Duggal, Sid Feldman, Corinne Fischer, Morris Freedman, Andrea Iaboni, Clement Ma, Krista L Lanctôt, Winnie Sun, Tarek K Rajji, Jennifer Watt, Frank Palmer, Gillian Strudwick, Peter Selby, Sanjeev Kumar

**Affiliations:** ^1^ Centre for Addiction and Mental Health, Toronto, ON, Canada; ^2^ Baycrest Hospital, Toronto, ON, Canada; ^3^ Toronto Dementia Research Alliance, Toronto, ON, Canada; ^4^ Ontario Shores Centre for Mental Health Sciences, Whitby, ON, Canada; ^5^ West Park Health Care Centre, Toronto, ON, Canada; ^6^ Baycrest Health Sciences, Toronto, ON, Canada; ^7^ Keenan Research Center for Biomedical Science, St. Michael's Hospital, Unity Health Network, Toronto, ON, Canada; ^8^ KITE Research Institute, Toronto Rehabilitation Institute ‐ University Health Network, Toronto, ON, Canada; ^9^ Sunnybrook Health Sciences Centre, Toronto, ON, Canada; ^10^ Ontario Tech University, Oshawa, ON, Canada; ^11^ Unity Health Toronto, Toronto, ON, Canada

## Abstract

**Background:**

Majority of long‐term care (LTC) residents experience cognitive impairment and behavioral and psychological symptoms of dementia (BPSD). Managing BPSD in LTC is often sub‐optimal and variable, resulting in inappropriate use of psychotropic medications and under‐utilization of non‐pharmacological interventions. We developed and tested an Integrated Care Pathway (ICP) that combines pharmacological and non‐pharmacological approaches for BPSD using a measurement based‐care approach in select psychiatry inpatient units and LTCs. We now aim to implement and evaluate the ICP combined with technology‐based monitoring systems and interventions (Tech‐ICP) in LTCs using pragmatic trial design.

**Method:**

Tech‐ICP is a technology‐driven solution that integrates assessment tools, monitoring systems, and algorithmic treatment based on ICP principles. It includes dashboards for the clinicians, wearable devices for biometric monitoring, and virtual reality (VR) interventions such as personalized reminiscence therapy and simulation‐based experiential learning for staff. We aim to implement the Tech‐ICP at 40 LTCs affiliated with academic hospitals across Toronto over three‐years in a step wedge design. The implementation will involve three phases, readiness assessment, implementation, and transition to sustainability. We will collect outcomes related to effectiveness of implementation, and site and individual level data related to clinical outcomes. The project will be co‐led by persons with lived experience to ensure alignment with priorities of LTC residents and caregivers.

**Result:**

This study will provide important insights regarding implementation of a clinical intervention for management of BPSD in LTC and its evaluation using pragmatic trial design. It is expected that the Tech‐ICP will enhance clinical team capabilities through technology to effectively treat BPSD, reduce inappropriate psychotropic medication use, improve quality of life of LTC residents, and reduce caregiver burden burnout. It is also expected to reduce rate of emergency room visits and hospital admissions for LTC residents.

**Conclusion:**

Tech‐ICP aims to provide a sustainable care model for managing BPSD in LTC's by integrating state of the art technological interventions. This approach is expected to improve care quality by ensuring appropriate use of non‐pharmacological and medications, and enhance the overall well‐being of both LTC residents and their caregivers. It will also inform design of future pragmatic trials in this population.

